# Regression of Sarcoidosis during Pregnancy: Case Report and Review of the Literature

**DOI:** 10.31138/mjr.31.4.416

**Published:** 2020-12-28

**Authors:** Senol Kobak

**Affiliations:** Istinye University Faculty of Medicine, LIV Hospital, Department of Internal Medicine and Rheumatology, WASOG Sarcoidosis Clinic, Istanbul, Turkey

**Keywords:** Sarcoidosis, pregnancy, regression

## Abstract

Sarcoidosis is a chronic granulomatous disease characterized by non-caseating granuloma formation. It usually involves the lung, but may also affects many organs and systems such as the musculoskeletal system, eye, skin, and heart. The data on the course of sarcoidosis during pregnancy are controversial. Generally, sarcoidosis patients do not have a decrease in fertility, and there is no increase in the incidence of congenital abnormalities or premature birth. On the other hand, sarcoidosis may progress during pregnancy. In this report, we discussed the effects of pregnancy on the disease activity in a patient with sarcoidosis.

## INTRODUCTION

Sarcoidosis is a chronic multisystemic disease characterized by non-caseating granuloma formation. Although its aetiology is not clear yet, it is probably considered to be a Th1 disease on a genetic background, that begins of the influence of environmental factors and infection agents.^1^ It often presents with lung involvement, but may affects many organs and systems such as the eye, skin, and heart. Sarcoidosis is a diagnosis of exclusion and it is based on clinical, radiological, laboratory and histopathological findings.^2^ It is a rare disease, with a prevalence of 4–65 / 100,000, which affecting more women than men. The disease is rare in pregnancy and its incidence is reported to be 0.02–0.06%.^3^ Data on the course and prognosis of the disease during pregnancy are limited and contradictory. Most of them are based on case reports and case series, rather than prospective studies (**Table 1**). Sarcoidosis shows three different radiological course during pregnancy: 1) normal chest radiographs before pregnancy, which remain the same during pregnancy; 2) stable residual disease, which remain stable during pregnancy; and 3) active disease improving during pregnancy.^4^

**Table 1.** Studies in the literature regarding sarcoidosis and pregnancy.

Patient age	Pregnancy	Features/involvement	Treatment	Mother outcome	Fetus outcome	Delivery (week)	Sarcoidosis diagnosis	References
33	18week	heart, lung, PAH	CS,digoxin	good	good	sectio/36week	BP	Euliano et al.
27	27week	Tx rejection/renal	CS	good	good	sectio/34week	BP	Kukura et al.
35	32week	cardiac arrest	CPR	death	death	sectio/32week	BP	Wallmüler et al.
20	18week	GN	CS	good	good	sectio/26week	BP	Warren et al.
28	postpartum	hypercalcemia	CS	good	good	sectio/36week	PP	Wilson-Holt et al.
27	postpartum	heart failure	CS	death	good	sectio/38week	PP/autopsy	Sebalos et al.
25	36week	hepatic/mesenteric sarcoidosis	CS	good	good	sectio/36week	BP	Vannoza et al.
37 31	17week 16week	heart sarcoidosis heart sarcoidosis	CS	good	good	Sectio/34week Sectio/32week	PP PP	Ertekin et al.
32	postpartum	arrhythmia/A-V block	CS,PM	good	good	sectio/36week	PP	Sugishita et al.
26	26week	dispnea/lung	CS	good	good	Sectio/38week	DP	Miloskovic et al.
33	12week	hypercalciuria	diet	good	good	Sectio/36week	BP	Subramanian et al.
25	28week	neurosarcoidosis	CS	good	good	Sectio/at term	DP	Cardonick et al.

PAH: pulmonary arterial hypertension; CS: corticosteroid; PM: pacemaker; A-V: atrioventricular; GN: granulomatous nephritis; Tx: transplantation; BP: before pregnancy; PP: postpartum; DP: during pregnancy.

Herein we reported the regression of disease during pregnancy and discussed the effects of pregnancy on disease course and prognosis in the light of the recent literature data.

## CASE REPORT

**Figure 1.**
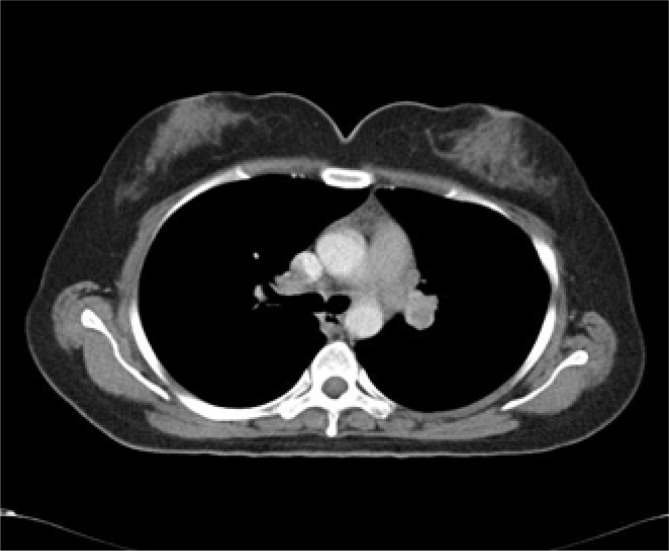
Thorax CT showed bilateral hilar lymphadenopathy (grade 1 sarcoidosis) – 3months after delivery.

A 32-year-old female patient which followed up in the rheumatology outpatient clinic with the diagnosis of sarcoidosis. One year ago, she was admitted with complaints of erythema nodosum, dry cough, effort dyspnoea, and ankle joint arthritis with 2-month duration. She had not reported any fever. On her medical history, she did not have any comorbid disease, was not a smoker, and had no family history of sarcoidosis. On laboratory tests; erythrocyte sedimentation rate (ESR): 45mm/h(normal<20mm/h); C-reactive protein (CRP): 16mg/dl (normal 0–5mg/dl); serum angiotensin converting enzyme (ACE): 62mg/dl (normal<45mg/dl) were detected. Serum calcium, liver, and renal function tests were normal. Bilateral hilar lymphadenopathy and interstitial changes were detected on thorax computed tomography. Endobronchial ultrasonographic (EBUS) biopsy was performed, and non-caseating granuloma was reported on pathological investigation. The patient was diagnosed with sarcoidosis according to clinical, radiological and histopathological findings. Other possible causes of granulomatous diseases (tuberculosis, bacterial, and fungal infections) were excluded. Pulmonary function tests (FVC:70%, DLCO:85%) were decreased and corticosteroid (CS) (methylprednisolone [MP]) 40mg/day and hydroxychloroquine (HQ) 400mg/day was started. Clinical, laboratory, and radiological regression was observed at the 6^th^ month of treatment and the dose of MP was reduced gradually to 4mg/day. At 12^th^ month the patient was admitted again with complaints of dyspnoea and dry cough. On her questionnaire, she said that she continued to receive MP 4mg/day and HQ 200mg/day. In laboratory tests; ESR: 54mm/h; CRP: 12.4 mg/dl, and serum ACE: 96mg/dl were detected. Complete blood count, liver and renal function tests and urinalysis were normal. She did not have any cardiac complaints, ECG was normal. On thorax CT, bilateral hilar lymphadenopathies and interstitial changes were reported. Pulmonary function tests were decreased. The patient was scheduled to start medium-high dose of MP and/or immunosuppressive drugs, but the patient refused these treatments. Three months later she came to the control visit, and said she was two months pregnant. As medication, she said that taken only MP 4mg/day and HQ 200mg/day. In laboratory tests; she had only iron deficiency anaemia; urinalysis, liver, and renal function test were normal. Serum ACE level was 64mg/dl; ESR: 38mm/h; CRP: 2.3mg/dl. Serum calcium and 1,25 hydroxy D3 were normal. Pulmonary function tests (FVC %82, DLCO=90) were better when compared with the old one. No radiological examination was performed due to pregnancy. During pregnancy the patient was followed up only with low dose MP 4mg/day and HQ 200mg/day. At 9^th^ month of pregnancy, the patient was delivered by Caesarean section, there were no complications in the infant and mother. Three months after delivery, she came to our outpatient clinic for control. She said that her complaints had regressed during pregnancy. Physical examination did not show any significant pathology. On laboratory investigations, acute phase reactants (ESR, CRP), renal and liver function tests, serum ACE level were normal (**Table 2**). Thorax CT revealed findings consistent with stage 1 sarcoidosis (**Figure 1**). Pulmonary function tests (FVC: 88%, DLCO: 92%) were reported as normal. An ophthalmologic examination revealed no retinopathy due to HQ use. The patient's general condition was good and the follow-up was continued.

**Table 2.** Laboratory findings, radiologic stage and pulmonary function tests of patient at initial diagnosis, at relapse, during pregnancy and after delivery.

**Findings**	**At initial diagnosis**	**At relapse**	**During pregnancy**	**After delivery**
ESR (normal<20mm/h)	45mm/h	54mm/h	38mm/h	12mm/h
CRP (normal 0-5mg/dl)	16mg/dl	12.4mg/dl	2.3mg/dl	4mg/dl
Serum ACE (normal<45mg/dl)	62mg/dl	96mg/dl	64mg/dl	23mg/dl
FVC	70%	76%	82%	88%
DLCO	85%	85%	90%	92%
Thorax CT-stage	Stage 2	Stage 2	No radiologic examination	Stage 1

ESR: erythrocyte sedimentation rate; CRP: C-reactive protein; ACE: angiotensin converting enzyme; FVC: force vital capacity; DLCO: diffusing capacity of the lung for carbon monoxide; CT: computed tomography.

## DISCUSSION

Herein, we reported the pregnant sarcoidosis patient which delivery a healthy infant. The patient's complaints regressed during pregnancy and improvement in pulmonary function tests was observed. Three months after delivery, thorax CT revealed radiological regression. Sarcoidosis is a multisystem granulomatous disease which aetiology is not clear yet. Literature data on the course and prognosis of the disease in pregnancy is still contradictory. According to some researchers, pregnancy does not affect the morbidity and mortality of the mother and child. In other words, the disease usually goes to remission during pregnancy and usually results in delivery of healthy babies. These researchers hypothesized that switching of Th1 to Th2 response occurs with the effect of oestrogen hormones, and weighted Th2 cytokines are secreted.^5^ The shift of immune response from Th1 cells to Th2 cells resulting in progressive increase of progesterone and oestrogen levels during pregnancy. Oestrogen stimulates Th2-mediated immunological responses, and increases the anti-inflammatory cytokines IL-4 and IL-10. Oestrogen also suppresses the production of Th1 cytokines, such as macrophage-attracting chemokines, IL-2, IL-12, and TNF-alpha. This is similar to other Th1 diseases such as rheumatoid arthritis or autoimmune thyroiditis. Sarcoidosis symptoms may improve during pregnancy, possibly due to increased levels of free cortisol in the circulation. Selroos et al. retrospectively evaluated 38 pregnancies in 252 female patients.^6^ In 25 of them, the disease appeared after birth, and the most common symptom was erythema nodosum. The other 13 patients had been followed up with known sarcoidosis diagnosis, and no complications were observed. The authors, who advocate opposing views, argue that sarcoidosis poses a risk to pregnancy, especially in patients with cardiac involvement. Therefore, it is inconvenient for these cases to become pregnant, or should follow-up closely during pregnancy. Complications such as miscarriages, preterm delivery, preeclampsia/eclampsia or foetal anomalies have been reported, but there is no increase of them compared to the normal population.^7^ Hadid et al. reported 678 sarcoidosis cases among 7 million births (prevalence 9.9/100.000 births) during 8-year period follow-up.^8^ They reported more preeclampsia, eclampsia, deep venous thrombosis, pulmonary embolism, and preterm birth in sarcoidosis patients. Wallmüller et al. reported a 35-year-old female patient with cardiac sarcoidosis.^9^ She was hospitalised with sudden cardiac arrest in the 32^nd^ week of pregnancy, but despite cardiopulmonary resuscitation, both the mother and the baby died. They recommended the use of antiarrhythmic drugs, CS, and pacemaker in such cases. Seballos et al. reported a 27-year-old pregnant patient with sarcoid cardiomyopathy detected in endomyocardial biopsy due to heart failure 5 days after birth.^10^ Despite treatment with CS, the patient died due to ventricular tachycardia and cardiac arrest. Cardiac sarcoidosis is an underdiagnosed disease that may be present in as many as 25% of patients with systemic sarcoidosis. Pregnant women with suspected cardiac involvement carry a considerable risk for a lethal cardiac event during pregnancy. However, not all patients with cardiac involvement had a poor prognosis during pregnancy. Euliano et al. reported 33-year-old pregnant sarcoidosis patient with pulmonary hypertension and cardiac involvement responded well to CS, digoxin, and anti-hypertensive drugs.^11^ Ertekin et al. reported arrhythmia as the first symptom in pregnancy in two cases.^12^ According to cardiac MRI and biopsy results, the diagnosis of sarcoidosis was made, and a healthy baby was born after treatment. Some studies showed that sarcoidosis is remitted in pregnancy, but may recur within the first 6 months after delivery. On other words, sarcoidosis behaves like a typical Th1 disease in pregnancy. Miyake et al. reported two cases of sarcoidosis with facial skin lesions appearing after delivery.^13^ Sugishita et al. reported complete atrio-ventricular block due to postpartum syncope episodes in a 32-year-old woman with sarcoidosis.^14^ They diagnosed cardiac sarcoidosis as a result of cardiac MRI and biopsy, and reported control of the disease with transient pacemaker and CS use.

Data on the treatment of sarcoidosis in pregnancy are insufficient in the literature. As spontaneous remission and/or stable disease is usually observed in pregnancy, most patients are followed up without medication. It is important to remember that some drugs (such as methotrexate, anti-TNF-alpha) are contraindicated during pregnancy. Corticosteroids are the first choice in patients with severe organ involvement (neurosarcoidosis, heart, kidney).^15^ It is possible to control the disease with moderate and/or high-dose CS. Sometimes as a steroid-sparing agent, as in our case, HQ may be preferable. Anti-arrhythmic drugs and transient or permanent pacemaker use have also been reported in patients with cardiac involvement. The efficacy of biologic drugs (especially TNF-alpha antagonists) in some severe form of sarcoidosis is well-known^16^; however, there is no data of the use of anti-TNF-alpha drugs in pregnant sarcoidosis patients.

## CONCLUSION

In conclusion, the data showing the impact of pregnancy on sarcoidosis course and prognosis are contradictory in the literature. Most of the studies supported the positive effect of pregnancy on the disease activity. However, there are also data showing that pregnancy can lead to disease progression. Prospective studies with a large number of patients are needed in this regard.
